# *Artemisia annua* Extract Improves the Cognitive Deficits and Reverses the Pathological Changes of Alzheimer’s Disease via Regulating YAP Signaling

**DOI:** 10.3390/ijms24065259

**Published:** 2023-03-09

**Authors:** Wenshu Zhou, Bingxi Lei, Chao Yang, Marta Silva, Xingan Xing, Hua Yu, Jiahong Lu, Wenhua Zheng

**Affiliations:** 1 Center of Reproduction, Development & Aging and Institute of Translation Medicine, Faculty of Health Sciences, University of Macau, Taipa, Macau SAR 999078, China; 2 State Key Laboratory of Quality Research in Chinese Medicine, Institute of Chinese Medical Sciences, University of Macau, Taipa, Macau SAR 999078, China; 3 Zhuhai UM Science & Technology Research Institute, Zhuhai 519000, China

**Keywords:** Alzheimer’s disease, *Artemisia annua*, neuroprotective effect, AD-type pathologies, YAP signaling pathway

## Abstract

Alzheimer’s disease (AD) is a chronic neurodegenerative disease characterized by the occurrence of cognitive deficits. With no effective treatments available, the search for new effective therapies has become a major focus of interest. In the present study, we describe the potential therapeutic effect of *Artemisia annua* (*A. annua*) extract on AD. Nine-month-old female 3xTg AD mice were treated with *A. annua* extract for three months via oral administration. Animals assigned to WT and model groups were administrated with an equal volume of water for the same period. Treated AD mice significantly improved the cognitive deficits and exhibited reduced Aβ accumulation, hyper-phosphorylation of tau, inflammatory factor release and apoptosis when compared with untreated AD mice. Moreover, *A. annua* extract promoted the survival and proliferation of neural progenitor cells (NPS) and increased the expression of synaptic proteins. Further assessment of the implicated mechanisms revealed that *A. annua* extract regulates the YAP signaling pathway in 3xTg AD mice. Further studies comprised the incubation of PC12 cells with Aβ_1–42_ at a concentration of 8 μM with or without different concentrations of *A. annua* extract for 24 h. Obtained ROS levels, mitochondrial membrane potential, caspase-3 activity, neuronal cell apoptosis and assessment of the signaling pathways involved was performed using western blot and immunofluorescence staining. The obtained results showed that *A. annua* extract significantly reversed the Aβ_1–42_-induced increase in ROS levels, caspase-3 activity and neuronal cell apoptosis in vitro. Moreover, either inhibition of the YAP signaling pathway, using a specific inhibitor or CRISPR cas9 knockout of YAP gene, reduced the neuroprotective effect of the *A. annua* extract. These findings suggest that *A. annua* extract may be a new multi-target anti-AD drug with potential use in the prevention and treatment of AD.

## 1. Introduction

Alzheimer’s disease (AD) is a chronic neurodegenerative condition with a worldwide incidence that is continuously increasing. Characterized by continuing memory loss and cognitive impairment, language disorders and functional and behavioral alterations, it has a dramatic bearing on patients’ quality of life [1]. Despite the increasing understanding of AD pathophysiology, so far, no effective drug has been proved successful against it, with available therapies failing to treat or prevent the disease progression [2]. AD pathological changes comprehend the presence of extracellular amyloid-β (Aβ) plaques and intracellular neurofibrillary tangles (NFTs) comprised of hyperphosphorylated microtubule-associated tau protein [3]. In AD brains, the overproduction and aggregation of Aβ peptides, tau hyperphosphorylation, increased oxidative stress, mitochondrial dysregulation, and microglial and astrocytic activation ultimately result in neuronal and synaptic loss, and in a significant atrophy in brain areas involved in the regulation of the cognitive function. Intracellular NFTs compromise both neuronal and synaptic functions and their extent and distribution have been reported to correlate with the duration and severity of AD [4].

Neurogenesis, comprising both the proliferation of neural stem or progenitor cells and the differentiation of neurons, is a long-term process responsible for maintaining the normal function of the central nervous system (CNS) [5]. Neurogenesis impairment in the brains of adult mice has been associated with learning and memory deficits, and other cognitive alterations [6]. In a recent study, an analysis of AD brains revealed a progressive decline of neurogenesis as the disease progressed in comparison with the brains of neurologically healthy individuals. Importantly, this decline manifests at early stages of the disease, before the presence of Aβ plaques and NFTs [7]. Therefore, the development of therapeutic strategies aiming to enhance adult neurogenesis could stand as promising novel treatment strategies to address AD.

The Hippo pathway is an evolutionary conserved kinase cascade, whose main components include the mammalian sterile 20-like 1/2 (MST1/2), large tumor suppressor homolog 1/2 (LATS1/2), MOB kinase activator 1A/B (MOB1A/B), Yes-associated protein (YAP) and TEAD family transcription factors [8,9]. Numerous evidence suggests that these components, especially YAP, have a key role in organ development and tissue homeostasis via regulation of cellular behaviors such as cell proliferation, survival, apoptosis, migration and differentiation [10]. Hippo signaling also controls stem cells self-renewal, proliferation and differentiation [11], triggering the regeneration of tissue [12]. Upon activation, MST1/2 kinase undergoes autophosphorylation and activates LATS1/2 and MOB1. LAST1/2 activation triggers the phosphorylation of YAP Ser127, promoting its cytoplasmatic retention and proteolytic degradation in a 14-3-3 protein-dependent manner [13]. Contrarily, inactivation of MST1/2 enables unphosphorylated YAP to translocate to the nucleus and bind to the TEA domain family (TEADs) transcriptional factors, triggering the transcription of genes involved in a series of biological occurrences, including cellular growth, proliferation and survival [14,15]. Many of these biological events are mapped with those involved in the pathogenesis of AD. In fact, the hyperactivity of Hippo cascade has been reported to be connected to Aβ-induced neuron death and amyloid precursor protein (APP) signaling in AD [12].

*Artemisia annua* (*A. annua*) herb has been used for more than 2000 years in traditional Chinese medicine being attributed different important biological properties including anti-malarial [16], anti-tumor [17,18], anti-inflammatory [19,20,21,22], antibacterial [23], antioxidant and immunoprotected effects [24,25,26]. Importantly, *A. annua* is non-toxic and harmless within a certain dose range [27], with several studies reporting its potential therapeutic use [28,29]. However, sesquiterpene lactones such as artemisinin, deoxyartemisinin and its derivatives are an important biologically active ingredient of artemisia annua *A. annua*. Among these, artemisinin has been the most extensively studied due to its widely recognized anti-malarial properties [30]. More recently, researchers reported the neuroprotective effect of artemisinin and its derivatives and their prospective use in the treatment of different neurodegenerative diseases [31,32]. Surprisingly, we found that soluble-water extract of *A. annua* was free of artemisinin when using the HPLC method. It contains deoxyartemisinic acid, artemisinin acid and arteether. Deoxyartemisinin, a compound without a peroxide bridge structure compared with artemisinin, was extracted from artemia annua. It demonstrated significant anti-inflammatory, antiulcer and other pharmacological effects [33]. More importantly, deoxyartemisinin showed better absorption, distribution, metabolism and excretion properties than artemisinin [33]. So far, there is no research on the neuroprotective effect of deoxyartemisinin on cognitive-related disease or AD, despite its promising clinical potential. Based on these findings, we speculate that *A. annua* might exert a neuroprotective effect that may be potentiated by the deoxyartemisinin, and arteether, and this effect might be modulated via regulation of YAP signaling. Therefore, the aim of this study was to investigate the neuroprotective effect of *A. annua* extract on AD and try to explore the potential underlying mechanisms of action. Then, *A. annua* was used as a compound to treat AD, which may provide important evidence to develop new drug for treatment AD. The obtained results showed that *A. annua* extract relieved AD symptoms and reversed the pathological changes of AD in vitro and in vivo via regulating the YAP pathway.

## 2. Results

### 2.1. A. annua Extract Improved the Cognitive Deficits of 3xTg AD Mice

**Figure 1:**
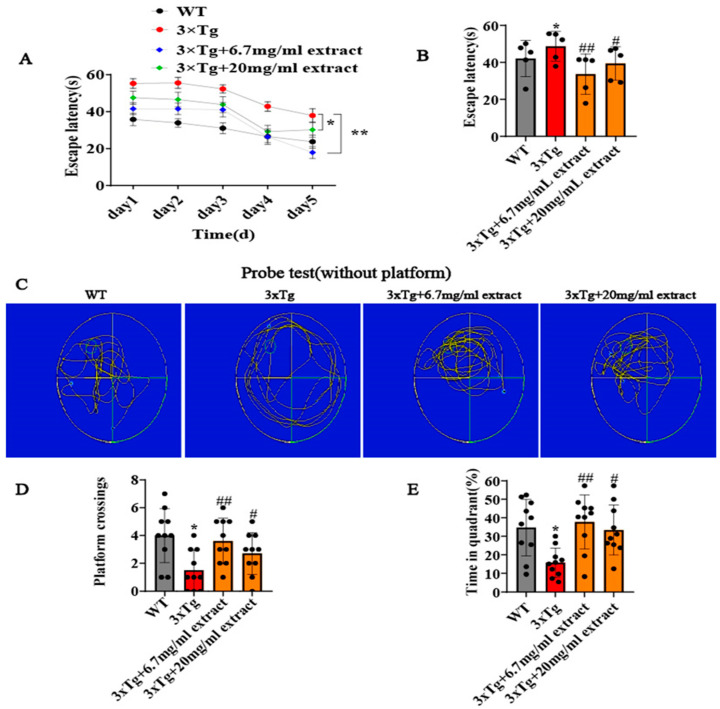
*A. annua* extract improved the behavioral performance of 3xTg AD mice. Morris water maze test results. (**A**) Escape latency during platform trials and (**B**) during spatial probe trial. (**C**) Representative image of the path of mice in 6 d trials. (**D**) Quantitative analysis of the number of platform crossings (**E**) and of the time spent in the quadrants (* *p* < 0.05 and ** *p* < 0.005; # *p* < 0.05 and ## *p* < 0.005 were considered statistically significant; ns was not considered significantly different. * Representative comparison between WT-Ctrl and 3xTg-Ctrl; # representative comparison between 3xTg-Ctrl and 3xTg+extract).

The potential of the extract of A. annua was obtained via different techniques (refer to Section 2) to improve the cognitive functions of 3xTg AD mice was assessed by Morris Water Maze (MWM) test. All mice were randomly divided into four groups: WT ctrl, 3xTg ctrl, the 20 mg/mL group and the 6.7 mg/mL group. After AD-type mice were treated different with concentrations of extract, except controls, which were treated with an equal volume of water, we found that the mice in the treatment groups performed better in the Morris water maze compared with 3xTg AD mice, as shown in Figure 1. Specifically, treated animals exhibited significant reductions in the escape latency (Figure 1A,B), denoting an improvement of their cognitive functions. Importantly, the escape latency of treated animals was similar to WT animals from the control group. *A. annua* extract treatment also promoted a significant increase in the number of platform crossings and of the percentage of time spent on the target quadrant (Figure 1C–E). These findings are indicative of the potential of extract to recover the learning and memory deficits of the mice in the 3xTg AD experimental model.

### 2.2. A. annua Extract Reduced Aβ Accumulation in 3xTg AD Mice

**Figure 2:**
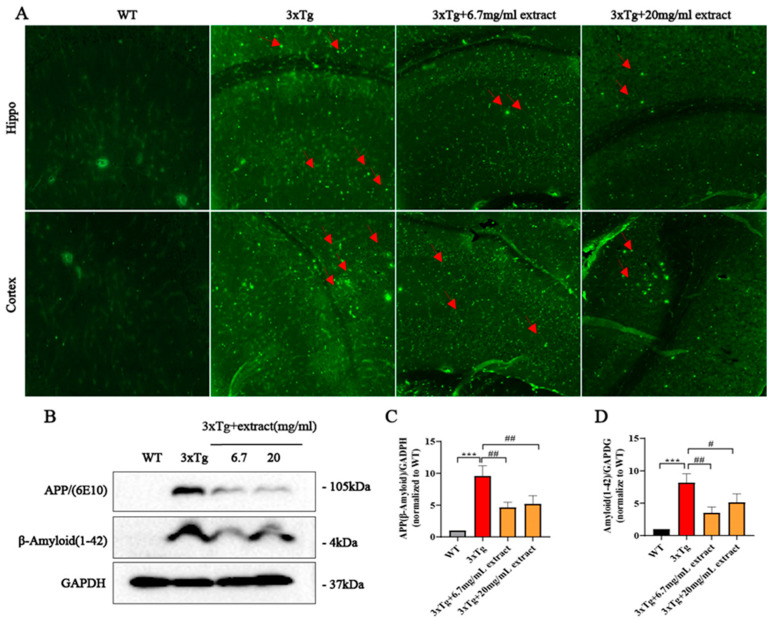
*A. annua* extract ameliorated amyloid deposition in 3xTg AD mice. (**A**) Representative images of β-amyloid(6E10) immunofluorescence staining in different brain areas (scale bars 50 μm). (**B**–**D**) Western blots and quantitative analysis of APP expression levels in brain homogenates. Each experiment was performed in triplicate. * Representative comparison between WT control and 3xTg AD untreated mice, # representative comparison between 3xTg AD groups; each assay was performed in triplicate. (*** *p* < 0.0005; # *p* < 0.05 and ## *p* < 0.005 were considered statistically significant; ns was not considered significantly different. * Representative comparison between 3xTg-Ctrl and WT-Ctrl; # representative comparison between 3xTg-Ctrl and 3xTg+extract).

Analysis of the effect of extract on Aβ plaques revealed that mice treated with extract exhibited a reduction in plaque burden in the brain cortex and hippocampus compared with untreated animals, as evaluated by 6E10 staining. Interestingly, the effect of extract on Aβ deposition was higher in animals treated with lower concentrations of the extract (Figure 2A). Further analysis of the expression of the amyloid precursor protein (APP) and Aβ_1–42_ in brain homogenates by western blot revealed that APP expression levels were significantly reduced in treated 3xTg AD mice in comparison with untreated 3xTg AD animals and, once again, this effect was more evident in the group treated with a lower extract dose (Figure 2B–D).

### 2.3. A. annua Extract Reduced Tau-Phosphorylation in 3xTg AD Mice

**Figure 3:**
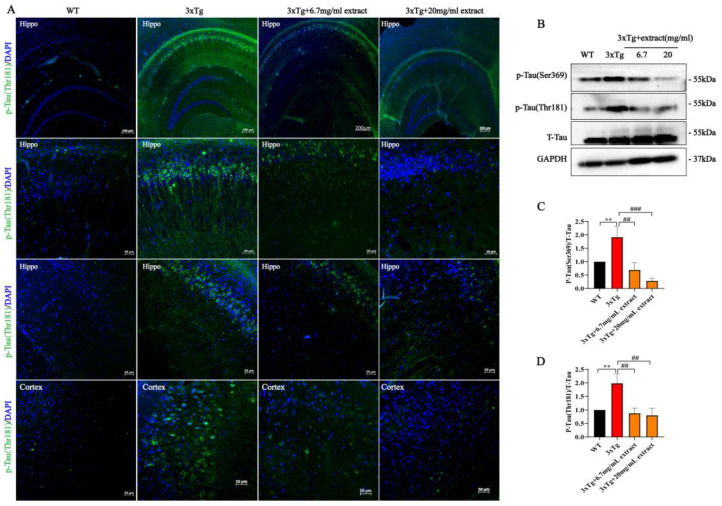
*A. annua* extract reduced Tau-phosphorylation in 3xTg AD mice. (**A**) Representative images of phosphorylated Tau immunofluorescence in the hippocampus and cortex of AD mice (scale bars 50 μM). (**B**–**D**) Western blot analysis and quantification of phosphorylated Tau at Ser369-Tau (p-Ser369) and Thr181-Tau (p-Thr181) sites, and total Tau (T-tau) in brain homogenates. (** *p* < 0.005; ## *p* < 0.005 and ### *p* < 0.0005 were considered statistically significant. * Representative comparison between WT-Ctrl and 3xTg-Ctrl; # representative comparison between 3xTg-Ctrl and 3xTg+extract).

Analysis of Tau phosphorylation showed that extract significantly improved Tau pathology (Figure 3). Specifically, the fraction area of Tau-Thr181-positive neurons in the subregions of the hippocampus and cortex of 3xTg AD-treated mice was significantly reduced in comparison with untreated mice (Figure 3A). Moreover, mice receiving the dosage of 20 mg/mL exhibited a more pronounced reduction of tau-phosphorylation compared with animals treated with 6.7 mg/mL (Figure 3A). Western blot analysis revealed that Tau-phosphorylation at different sites (Serine369 and Threonine181) was significantly reduced in the brain of 3xTg AD-treated mice groups. Interestingly, we also found that the animals treated with 20 mg/mL dose presented a more obvious decrease in Tau-phosphorylation (Figure 3B–D). Still, the herbal extract did not affect the expression of total-Tau.

### 2.4. A. annua Extract Attenuated Neuroinflammation in 3xTg AD Mice

**Figure 4:**
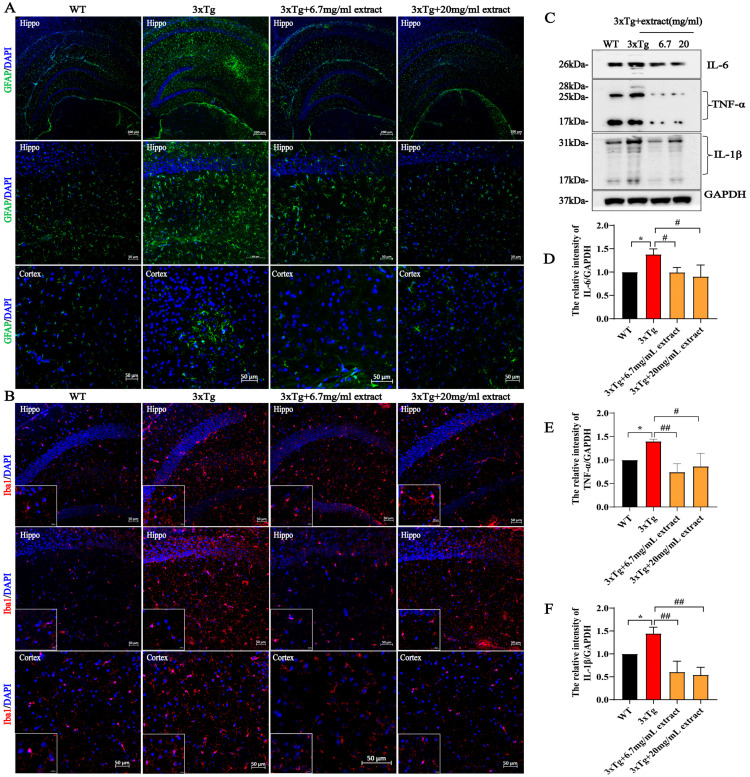
*A. annua* extract attenuated neuroinflammation in 3xTg AD mice. (**A**,**B**) GFAP and Iba-1 immunostaining in the hippocampus and cortex of 3xTg AD-treated and untreated mice (scale bars 50 μM). (**C**) The expression of the inflammatory factors IL-6, TNF-α and IL-1β, in brain homogenates was detected by Western blot. (**D**–**F**) Quantification of western blot from (**C**). Each assay was performed in triplicate. (* *p* < 0.05; # *p* < 0.05 and ## *p* < 0.005 were considered statistically significant. * Representative comparison between WT-Ctrl and 3xTg-Ctrl; # representative comparison between 3xTg-Ctrl and 3xTg+extract).

Increasing evidence suggests that the occurrence of neuroinflammatory alterations, such as chronic microgliosis and astrogliosis, are crucial contributors to the progression of AD pathology [34,35]. Therefore, we evaluated the expression of Glial fibrillary acidic protein (GFAP, astrocytes marker) and Iba-1 (microglia marker) in the hippocampus and cortex. The obtained results indicate that the expression of GFAP was significantly increased in 3xTg AD mice in comparison with WT mice, being significantly reduced by extract treatment (Figure 4A). Likewise, untreated 3xTg AD mice exhibited higher Iba-1 expression levels compared with WT mice, being reduced in both the 6.7 mg/mL and 20 mg/mL treatment groups (Figure 4B). To further support these findings, the levels of IL-6, TNF-α and IL-1β were assessed by western blot. Compared with untreated 3xTg AD, mice in both the 6.7 mg/mL and 20 mg/mL treatment groups showed a significant reduction of the expression levels of these inflammatory factors (Figure 4C–F).

### 2.5. Extract Promoted the Proliferation of Neural Progenitor Cells and Increased the Expression Synaptic Proteins in 3xTg Mice

**Figure 5:**
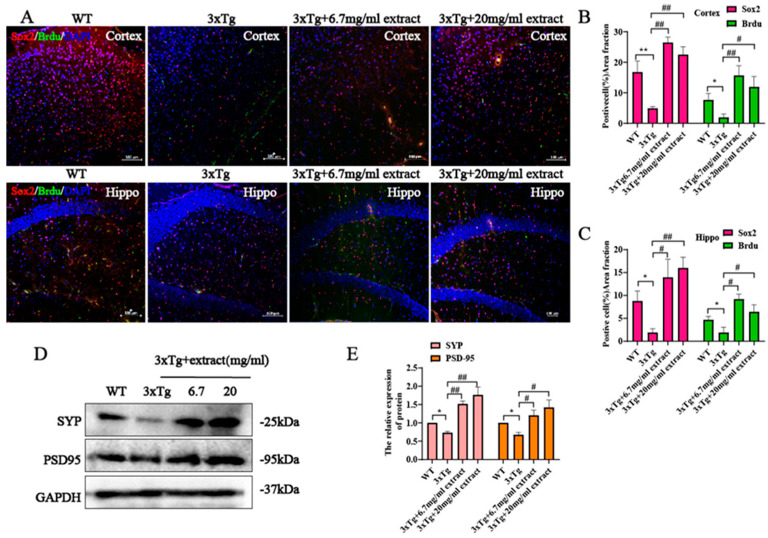
*A. annua* extract promoted the proliferation of neural progenitor cells and increased synaptic plasticity in 3xTg AD mice. (**A**) Representative images of Sox2+ and Brdu+ in the hippocampus and cortex (scale bars 100 μM), (**B**,**C**) Quantification of the number of Sox2+ and Brdu+ cells. (**D**) Western blot analysis of synapse-related protein including synaptophysin (SYP) and PSD95 in brain homogenates. (**E**) Quantification of western blot from (**D**). (* *p* < 0.5 and ** *p* < 0.005; # *p* < 0.05 and ## *p* < 0.005 were considered statistically significant. * Representative comparison between WT-Ctrl and 3xTg-Ctrl; # representative comparison between 3xTg-Ctrl and 3xTg+extract).

Evidence suggests the occurrence of neurogenesis impairments in the AD. The decline of adult neurogenesis that accompanies AD progression has been associated with an aggravation of the cognitive deficits and its upregulation has been shown to improve cognition [36]. Hence, the development of treatments able to enhance neurogenesis are a potential therapeutic approach to be used in the treatment of AD. Immunofluorescence double-labeling of neuronal progenitor cells (NPs) with Sox2 and Brdu (marker of proliferative cells) in the cortex and hypothalamus showed a significative reduction of the number of Sox2+ and Brdu+ positive cells in 3xTg AD mice compared with WT control animals (Figure 5A). In contrast, 3xTg AD mice treated with extract exhibited a significative higher number of Sox2^+^and Brdu^+^-positive cells in the same brain areas (Figure 5A). Additionally, the number of Sox2^+^ and Brdu^+^-positive cells was determined compared with untreated 3xTg AD mice; treated mice exhibited significant increased numbers of Sox2+ and Brdu+ positive cells in both the 6.7 mg/mL and 20 mg/mL treatment groups (Figure 5B,C), indicating that extract promoted the survival and proliferation of NPs in 3xTg AD mice. Further investigation of the effect of extract in the dendritic loss exhibited by 3xTg AD mice showed that both 6.7 mg/mL and 20 mg/mL doses of extract treatment promoted the upregulation of synapse-related protein expression levels (Figure 5D,E), indicating that extract rescued the dendritic loss in the brains of 3xTg AD mice.

### 2.6. Extract Rescued Neuronal Cell Apoptosis in 3xTg AD Mice via Regulating Hippo/YAP Signaling

**Figure 6:**
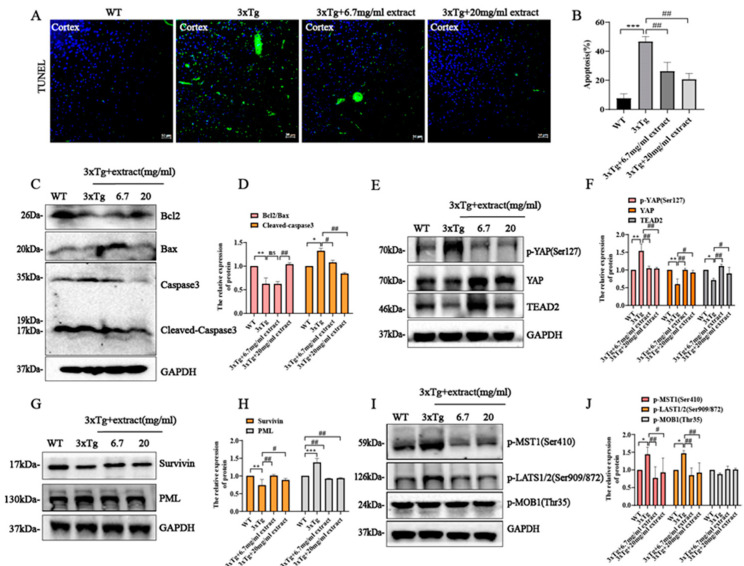
*A. annua* extract rescued neuronal cell apoptosis in 3xTg AD mice via regulating Hippo/YAP signaling. (**A**) Apoptosis determined by TUNEL staining in the cortex (scale bars 50 μm). (**B**) Quantification of western blot form (**A**). (**C**) The expression of Bax, Bcl2, caspase 3 and cleaved caspase-3 and GAPDH was detected by Western blot. (**D**) Quantification of western blot from (**C**). (**E**) Western blot was used to assess the expression of p-YAP, YAP, EDTA2, GAPDH. (**F**) Quantification of western blot from (**E**). (**G**) The downstream proteins expression of YAP such as Survivin and PML was detected by western blot. (**H**) Quantification of western blot from (**G**). (**I**) The upstream proteins expression of YAP including p-MST1(Ser410), p-LAST1/2(Ser909/872), p-MOB1(Thr35) and GAPDH was assessed by western blot. (**J**) Quantification of western blot from (**I**) (* *p* < 0.05, ** *p* < 0.005 and *** *p* < 0.0005; # *p* < 0.05 and ## *p* < 0.005 were considered statistically significant; ns was not considered significantly different. * Representative comparison between 3xTg-Ctrl and WT-Ctrl; # representative comparison between 3xTg-Ctrl and 3xTg+extract).

Multiple evidence indicates that the Hippo signaling pathway plays a critical role in neurodegeneration disease [37]. Moreover, in a recent study, YAP was also implicated as a hub molecule in AD pathology [38]. Assessment of the effect of extract in the neuronal apoptosis of AD mice by TUNEL staining assay indicates that the increased number of apoptotic cortical neurons in 3xTg AD mice was significantly decreased by extract treatment (Figure 6A,B). These alterations were accompanied by changes in the expression of different apoptosis regulators including Bax, Bcl-2 and cleaved caspase 3 (Figure 6C). Specifically, extract promoted the increase in Bcl-2/Bax ratio and the decrease in cleaved caspase 3 expression (Figure 6D). Previous studies indicate the involvement of the Hippo signaling in the inhibition of apoptosis. Activation of the Hippo signaling pathway triggers the functional inactivation of YAP, promoting the occurrence of cellular apoptosis [14,36]. Accordingly, the expression levels of YAP and TEAD2 were significantly increased in treated 3xTg AD mice compared with untreated 3xTg AD animals, while YAP phosphorylation levels were significantly decreased by extract treatment (Figure 6E,F). Previous studies suggest that YAP interaction with TEAD transcription factors may result in the upregulation of anti-apoptotic genes such as Survivin [39,40,41] On the other hand, phosphorylated YAP may also bind to p73, promoting the upregulation of pro-apoptotic genes such as bax and promyelocytic leukemia (PML) [42,43]. In this study, extract treatment promoted a significant increase in Survivin expression levels while significantly reducing the expression of PML (Figure 6G,H). In addition, the proteins levels of upstream p-MST1(Ser410) and p-LATS1/2(Ser909/872) of the YAP signaling cascade were significantly decreased by extract treatment, while p-MOB1(Thr35) expression was not significantly altered (Figure 6I,J). These results indicate that extract reduction of brain neuronal apoptosis may occur via YAP/TEAD2/Survivin signaling.

### 2.7. A. annua Extract Antagonized Aβ_1–42_-Induced Neurotoxicity and Increased the Viability of Neuronal Cells In Vitro

**Figure 7:**
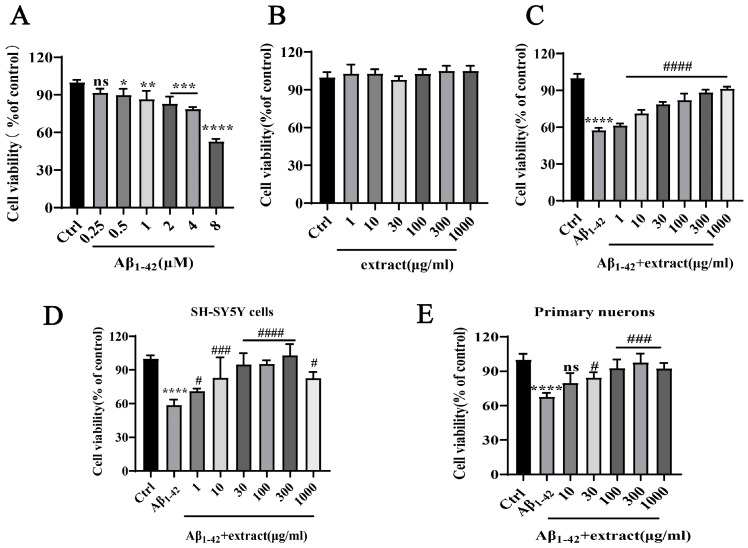
*A. annua* extract reduced the neurotoxicity triggered by Aβ_1–42_. (**A**) Assessment of Aβ_1–42_ cytotoxicity. Cells were treated with different concentrations of Aβ_1–42_ (0.5–8 μM) for 24 h and cell viability was assessed using the MTT assay. (* *p* < 0.05, ** *p* < 0.005, *** *p* < 0.0005, **** *p* < 0.0001 were considered as statistically significant, * representative comparison between Ctrl and Aβ_1–42_, ns was not considered significantly different). (**B**) Assessment of extract neurotoxicity. Cells were treated with different concentrations of extract (1–1000 μg/mL) for 24 h and cell viability was measured using the MTT assay. (**C**) Assessment of the effect of extract treatment on Aβ_1–42_-induced neurotoxicity. Cells were incubated with 8 μM Aβ_1–42_ and treated with different concentrations of extract for 24 h and cellular viability was assessed using the MTT assay. (**D**) Cellular viability of SH-SY5Y cells treated with 8 μM Aβ_1–42_ alone or with (1~1000 μg/mL) extract. (**E**) Cellular viability of primary neurons treated with 8 μM Aβ_1–42_ alone or with (1~1000 μg/mL) extract. Each assay was performed in triplicate and the experiment was repeated three times. (**** *p* < 0.0001; # *p* < 0.05, ### *p* < 0.0005 and #### *p* < 0.0001 were considered statistically significant; ns was not considered significantly different. * Representative comparison between Ctrl and Aβ_1–42_; # representative comparison between Aβ_1–42_ and extract).

Aiming to assess the neurotoxic effect of Aβ_1–42_ in vitro, PC12 cells were incubated with increasing concentrations of Aβ_1–42_ for 24 h. Cell viability results revealed that incubation with Aβ_1–42_ had a significant cytotoxic impact at a concentration of 8 μM, resulting in the death of 50% of neuronal cells (Figure 7A). The study of the potential protective action of *A. annua* against Aβ_1–42_-induced toxicity was preceded by the evaluation of the effect of the different extract against Aβ_1–42_. Incubation of PC12 cells with different concentrations of extract (1–1000 μg/mL) did not induce cytotoxicity on neuronal cells (Figure 7B). Incubation of PC12 cells with different concentrations of extract and 8 μM Aβ_1–42_ for 24 h revealed that the extract increased the viability of cells in a dose-dependent manner, starting at the concentration of 10 μg/mL (Figure 7C). Similar results were obtained using human neuroblastoma SH-SY5Y cells and primary cortical neurons, in which extract dose-dependently protected these cells from Aβ_1–42_-induced neurotoxicity (Figure 7D,E). These results propose that extract has a strong inhibitory action against the neurotoxicity of Aβ_1–42_ exerting a neuroprotective action.

### 2.8. Extract Treatment Alleviated Aβ_1–42_-Induced Apoptosis by Aβ_1–42_ in PC12 Cells

**Figure 8:**
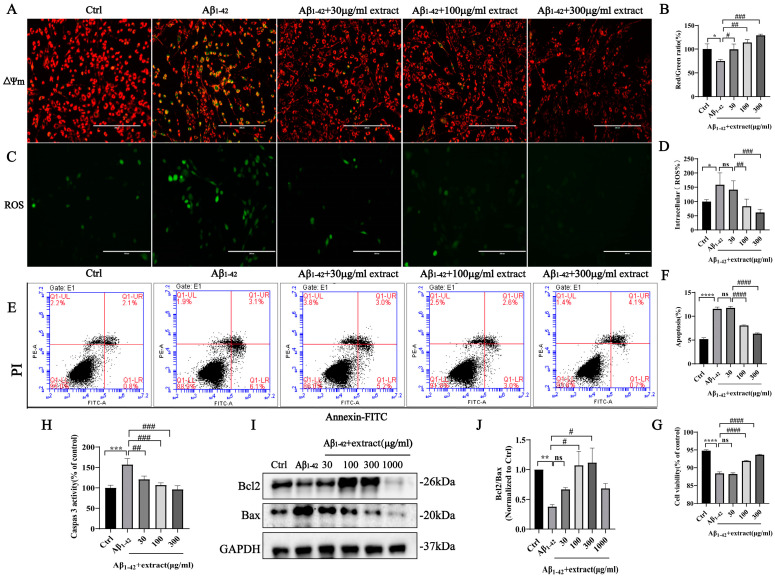
*A. annua* extract attenuated apoptosis induced by Aβ_1–42_ in PC12 cells. PC12 cells were treated with 8 μM Aβ_1–42_ alone or with (30 μg/mL, 100 μg/mL, 300 μg/mL) extract. (**A**,**B**) The restoration of the mitochondrial membrane potential was denoted by the shift of red fluorescence to green indicated by JC-1 staining. (**C**,**D**) Intracellular ROS levels were measured by the CellROXs Deep Green Reagent (scale bars 200 μm). (**E**–**G**) Flow cytometry and quantitative analysis of cellular apoptosis. (**H**) Caspase 3 was detected by caspase 3 activity test assay kit. (**I**) Western blot analysis of Bcl2 and Bax and GAPDH. (**J**) Quantification of Bcl2/Bax expression from western blot form (**I**). (* *p* < 0.05, ** *p* < 0.005, *** *p* < 0.0005 and **** *p* < 0.0001; # *p* < 0.05, ## *p* < 0.005, ### *p* < 0.0005 and #### *p* < 0.0001 were considered statistically significant; ns was not considered significantly different. * Representative comparison between Ctrl and Aβ_1–42_; # representative comparison between Aβ_1–42_ and extract).

Apoptosis is a key event in AD, with studies reporting that Aβ can directly trigger apoptotic neuronal death in vitro and in vivo [44,45]. Moreover, evidence suggests the upregulation of several cell-death regulatory proteins in AD brains [46,47]. In this study, extract promoted the decrease in Aβ_1–42_-induced increase in PC12 cell apoptosis as shown in Figure 8. Incubation of PC12 cells with 8 μM Aβ_1–42_ promoted a significative impairment of the mitochondrial function denoted by the decrease in Δψm that was prevented by extract treatment (Figure 8A,B). Further evaluation of ROS production revealed a significant increase in intracellular ROS levels induced by Aβ_1–42_ that was reduced by extract treatment (Figure 8C,D). Additionally, flow cytometry results showed that extract acted against Aβ_1–42_-induced cell apoptosis (Figure 8E,F). In addition, the increase in caspase 3 activity (Figure 8H) and decrease in the expression ratio between the anti-apoptotic Bcl2 and pro-apoptotic Bax proteins promoted by Aβ_1–42_ incubation was reversed by extract treatment (Figure 8I,J). Similar results were obtained using human neuroblastoma SH-SY5Y cells and primary neurons (Supplementary Figure S1), suggesting that the extract has a potent anti-apoptosis effect.

### 2.9. YAP Involved in the Protective Effect of A. annua Extract in PC12 Cells

**Figure 9:**
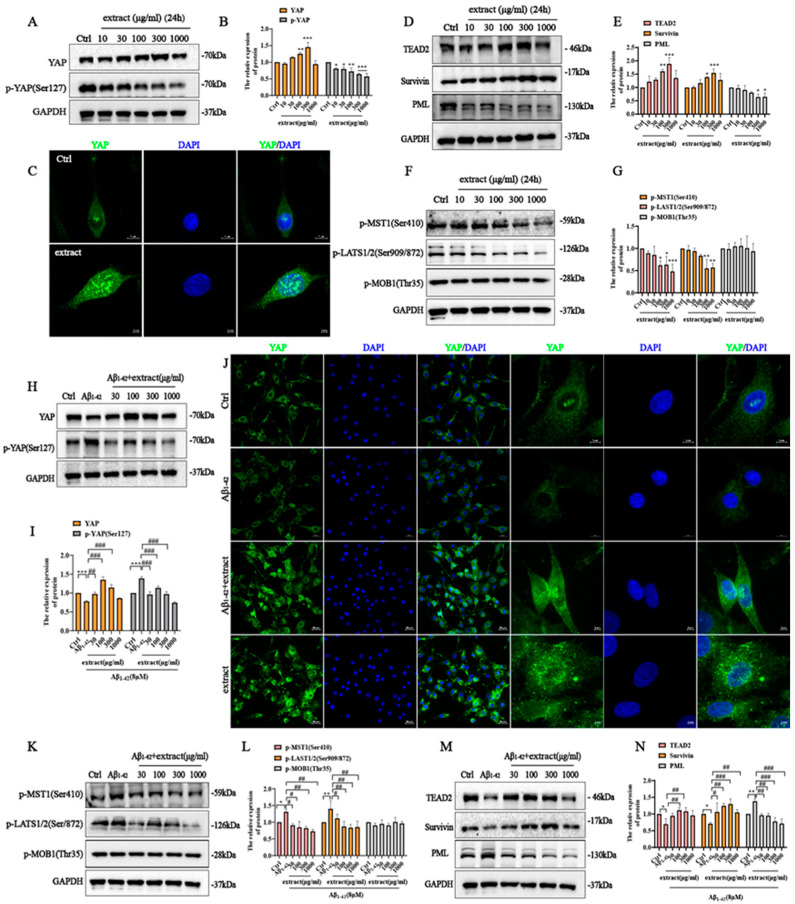
*A. annua* extract’s protective effect occurs via regulating YAP signaling in PC12 cells. PC12 cells were treated with (30 μg/mL, 100 μg/mL, 300 μg/mL and 1000 μg/m) extract for 24 h. (**A**) Western blot analysis of YAP, P-YAP2 and GAPDH expression. (**B**) Quantification of A, extract increased the expression of YAP and decreased the phosphorylation of YAP in a dose-dependent manner. (**C**) Immunofluorescence of YAP (scale bars 5 μm). (**D**) The expression of TEAD2, Survivin, PML and GAPDH were detected by western blot. (**E**) Quantification of western blot from (**D**), extract promoted the expression of TEAD2 and Survivin in PC12 cells, and decreased the expression of PML. (**F**) Western blot analysis of p-MST1(Ser410), p-LATS1/2(Ser909/872) and p-MOB1(Thr35) expression. (**G**) Quantification of western blot from (**F**), extract inhibited the phosphorylation of MST1 and LATS1/2, while no effect the expression of p-MOB1(Thr35). (* *p* < 0.05, ** *p* < 0.005 and *** *p* < 0.0005 were considered statistically significant. * Representative comparison between Ctrl group and extract groups). (**H**) PC12 cells were incubated with 8 μM Aβ_1–42_ with or without extract at different concentrations for 24 h. Western blot analysis of the effect of extract on YAP, p-YAP and GAPDH. (**I**) Quantification of western blot from (**H**). (**J**) Immunofluorescence of YAP expression (scale bars 20 μm and scale bars 5 μm). (**K**) Western blot analysis of p-MST1(Ser410), p-LATS1/2(Ser909/872), p-MOB1(Thr35) and GAPDH expression. (**L**) Quantification of western blot from (**K**). (**M**) Western blot analysis of TEAD2, Survivin, PML and GAPDH. (**N**) Quantification of western blot from (**M**). (* *p* < 0.05, ** *p* < 0.005 and *** *p* < 0.0005; # *p* < 0.05, ## *p* < 0.005 and ### *p* < 0.0005 were considered statistically significant; ns was not considered significantly different. * Representative comparison between Ctrl and Aβ_1–42_, # representative comparison between Aβ_1–42_ and extract).

Assessment of the potential involvement of the Hippo/YAP signaling on the protective effect of extract in PC12 cells showed that extract treatment promoted a dose-dependent increase in YAP and a downregulation of YAP phosphorylation levels (Figure 9A,B). ICC assay further confirmed that extract treatment promoted YAP nuclear translocation and a significant increase in its expression levels (Figure 9C), subsequently driving an increase in TEAD2 (Figure 9D,E). In addition, extract treatment also promoted a dose-dependent increase in Survivin expression and a decrease in PML expression, which are two downstream anti- and pro-apoptosis genes of YAP signaling, indicating that extract anti-apoptotic effect may occur, at least in part, via regulation of Hippo signaling. (Figure 9D,E). Further study found that extract reduced the increase in p-MST1(Ser410) and p-LAST1/2 (Ser909/872) expression while having no significant effect in p-MOB1(Thr35), which are located upstream of the YAP signaling cascade (Figure 9F,G). Together with these findings suggest that Hippo/YAP pathway may be involved in regulating the protective effect of extract.

To investigate this hypothesis, we checked the alterations in the Hippo signaling upon incubation with Aβ_1–42_ and treatment with extract. The obtained results showed a decrease in YAP expression upon Aβ_1–42_ incubation that was gradually increased by 300 μg/mL extract treatment (Figure 9H,I). In contrast, Aβ_1–42_ promoted the upregulation of YAP phosphorylation that was attenuated by extract treatment (Figure 9H,I). ICC assay further confirmed that extract promoted a significant increase in YAP expression and its translocation to the nucleus (Figure 9J). Aβ_1–42_ also promoted the upregulation of p-MST1(Ser410) and p-LATS1/2 (Phospho-Ser909/872) expression and these were altered by extract treatment. No significant changes were identified on p-MOB1(Thr35) of Hippo signaling (Figure 9K,L). TEAD2 was significantly downregulated upon incubation with Aβ_1–42_ and successfully rescued by extract treatment (Figure 9M,N). In addition, the decrease in Survivin and increase in PML expressions caused by Aβ_1–42_ incubation was attenuated by extract treatment (Figure 9M,N). These findings show evidence that extract is able to protect against Aβ_1–42_-induced apoptosis via regulation of YAP signaling.

**Figure 10:**
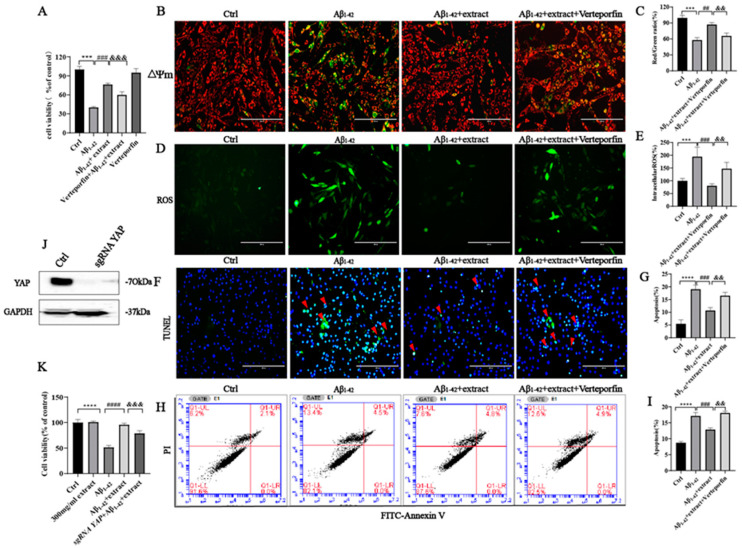
YAP signaling mediated the protective effects of *A. annua* extract in PC12 cells. PC12 cells were pretreated with 2.5 μM Verteporfin (YAP inhibitor) for 60 min and incubated with 8 μM Aβ_1–42_ in the presence or absence of 300 μg/mL extract. (**A**) Verteporfin inhibited the protective effect of extract on Aβ_1–42_-induced neurotoxicity. (**B**,**C**) The mitochondrial membrane potential was measured by JC-1 staining (scale bars 200 μM). (**D**,**E**) Intracellular ROS levels were measured by ROS assay (scale bars 200 μM). (**F**,**G**) Apoptosis was measured by TUNEL staining (scale bars 200 μM). (**H**,**I**) Apoptosis was measured by Flow cytometry. (**I**) Cell viability was measured by MTT assay. (**J**) Western blotting analysis of YAP expression in WT and KO cells. (**K**) The viability of cells was measured by MTT assay, MTT assay was performed to assess the viability of cells in WT and KO cells. Each experiment was repeated in triplicate. (*** *p* < 0.0005 and **** *p* < 0.0001; ## *p* < 0.005, ### *p* < 0.0005 and #### *p* < 0.0001; && *p* < 0.005 and &&& *p* < 0.0005 were considered statistically significant; ns was not considered significantly different. * Representative comparison between Ctrl and Aβ_1–42_, # Representative comparison between Aβ_1–42_ and extract; representative comparison between Aβ_1–42_ and Aβ_1–42_+extract+Verteporfin).

To further verify whether YAP is associated with the survival effect promoted by extract against Aβ_1–42_-induced cell apoptosis, the cells were pretreated with verteporfin (a specific inhibitor of YAP) for 60 min at a concentration of 2.5 μM [48] followed by incubation with extract and Aβ_1–42_ for 24 h. MTT assay results showed that verteporfin at a concentration of 2.5 μM blocked the neuroprotective action of extract against Aβ_1–42_-induced neurotoxicity (Figure 10A). Incubation of cells with verteporfin for 60 min prior to Aβ_1–42_ alone or with extract prevented the recovery of the mitochondrial membrane potential (Figure 10B,C) and the decrease in intracellular ROS levels promoted by extract (Figure 10D,E). TUNEL staining and flow cytometry results showed that verteporfin also blocked the inhibitory effect of extract against Aβ_1–42_-induced cell apoptosis (Figure 10F–I). YAP knockout using Crispr Cas9 approaches further confirmed the involvement of YAP signaling on the extract’s neuroprotective effect. After confirmation of the successful knockout of YAP on PC12 cells by Western blot analysis (Figure 10J), the cells were incubated with 8 μM Aβ_1–42_ with or without extract for 24 h. MTT assay results confirmed that the neuroprotective effect of extract was blocked in the KO cellular model (Figure 10K), further validating the involvement of YAP signaling in the protective effects of extract against Aβ_1–42_-induced toxicity.

### 2.10. HPLC Analysis of A. annua Extract Powder

*A. annua* extract was successfully extracted from *A. annua* and was found to have beneficial effects in both in vitro and in vivo experiments against AD, which is likely to be attributed to synergistic actions of several of its constituents.

The extract characterization by HPLC analysis identified artemisinin acid, arteether and deoxyartemisinin as three of its main active components, while no artemisinin was found (Supplementary Figure S2A–D). Subsequently, an MTT assay was performed to compare the neuroprotective effects of extract, deoxy-artemisinin, arteether and artemisinin acid in the Aβ_1–42_-induced PC12 cell model, respectively. Results from MTT assay showed that extract, deoxyartemisinin and arteether exhibited neuroprotective properties, while having no neuroprotective effect in artemisinin acid (Supplementary Figure S2E,F). These findings suggest that neuroprotective effect of extract in vivo and in vitro may be a result of synergistic actions of arteether and deoxyartemisinin.

## 3. Discussion

AD is an intricate multifactorial disease caused by an interaction between genetic and environmental factors [49]. Despite extensive research, there is still no effective treatment able to halt the development or progression of this disease, whose worldwide incidence has been continuously increasing. Therefore, we propose that a therapeutic approach able to target multiple pathways to stop the different signaling cascades driving AD pathogenesis including Aβ aggregation, Tau phosphorylation, oxidative stress and mitochondrial dysfunction could pose as an effective strategy. *A. annua*-derived compound artemisinin and its analogue artemether have been associated with a broad spectrum of neuroprotective actions, holding encouraging prospects for future AD therapies [50,51]. However, the potential therapeutic effect of *A. annua* on the cognitive impairments and pathological changes observed during AD are still not known. In the present study, we described for the first time the potential of the extract to significantly improve 3xTg AD mice’ cognitive deficits, reduce Aβ accumulation, hyper-tau-phosphorylation and the release of inflammatory and apoptotic factors. Moreover, the extract promoted the survival and proliferation of neural progenitor cells (NPs), increased the expression of synaptic proteins and inhibited neuronal cell death while stimulating the activation of the Hippo signaling pathway. In this study, we also reported that extract was able to protect neuronal cells against the neurotoxicity induced by Aβ_1–42_ by promoting cell survival, restoring the mitochondrial membrane potential loss, inhibiting ROS overproduction and attenuating Aβ_1–42_-induced apoptosis. It also stimulated the increase in YAP expression while decreasing YAP phosphorylation, suggesting the involvement of the Hippo/YAP signaling pathway in the mediation of its protective effects.

Transgenic 3xTg AD mice develop many of the AD hallmarks, including Aβ and tau pathology, neuroinflammation and cognitive deficits [52]. In this study, oral administration of extract to 3xTg AD mice for 3 months significantly improved their learning and memory deficits as denoted by the increased escape latency, number of platform crossings and time spent on the target quadrant in the Morris water maze test. Further testing also revealed an important impact of the treatment on Aβ aggregation and tau hyperphosphorylation by promoting a significative decrease in its expression levels in the cerebral cortex and hippocampus. There are two possible reasons to explain this occurrence. The first is that *A. annua* extract may have inhibited the continuingly increase in oligomers and plaques caused by Aβ by interfering with the activity of β-secretase 1 or γ-secretase. Another possibility is that the plaques that were already formed may have been cleared by microglia after the treatment. These possibilities need to be further explored in future studies. Increasing evidence suggest that the occurrence of neuroinflammatory changes, including chronic microgliosis and astrogliosis are also key contributors to the progression of AD pathology [53,54]. As resident immune cells of the central nervous system (CNS), microglia promote Aβ clearance in the early stages of the disease hinder its progression [55]. However, microglia overactivation triggers the release of different pro-inflammatory factors, contributing to the installation of a neuroinflammatory state in AD brains [56,57]. Similarly, astrocytes also play a key role in AD progression with evidence suggesting the accumulation of reactive astrocytes around amyloid plaques contributing to scar formation [57]. Our results showed that extract promoted an improvement of the animals’ neuroinflammatory state by inducing a significant decrease in astrocytes and microglia expression and ultimately impairing the release of different pro-inflammatory factors including IL-1β, IL-6 and TNF-α. 

The neurotoxicity of Aβ_1–42_ has been widely reported with different studies describing the use of this peptide fragment in the development of AD in vitro experimental models [58]. In line with previous reports, the incubation of neuronal cells with increasing concentrations of Aβ_1–42_ promoted a dose-dependent cytotoxic effect denoted by the decrease in cellular viability [59,60]. Treatment with extract efficiently suppressed Aβ_1–42_ neurotoxicity by promoting the survival of different neuronal cells, including PC12 and SH-SY5Y cell lines and primary neurons. Further assessment of the damage induced by Aβ_1–42_ incubation revealed the occurrence of mitochondrial membrane potential loss, increase in ROS production and apoptosis. Widely recognized as key players in the course of AD progression, the occurrence of mitochondrial dysfunction, oxidative damage and apoptosis was prevented by extract. Moreover, it also prevented the upregulation of many of the pathological and pro-inflammatory markers promoted by Aβ_1–42_ incubation. These findings provide evidence of extract potential treatment effect against AD using in vivo and in vitro experimental models.

Several core components of the Hippo signaling pathway, especially YAP, have been reported to play a crucial role in neurodegenerative disease, being involved in the regulation of neural cellular apoptosis [37], and YAP has been recently implicated as a hub molecule in AD pathology [38,61]. Likewise, this study showed evidence of the targeting potential of this pathway in AD prevention and treatment. Assessment of the mechanisms underlying extract protective action against the occurrence of neuronal death in vivo revealed that it is mediated, at least in part, via regulation of YAP signaling. The brains of mice treated with the herbal extract exhibited an accentuated decrease in the number of apoptotic cortical neurons and alterations in the expression of different apoptosis regulators including Bax, Bcl-2, cleaved caspase 3, Survivin and PML. These changes were accompanied by an increase in YAP and TEAD2 expression and a decrease in p-YAP(Ser127), p-MST1(Ser410) and p-LATS1/2(Ser909/872). Similarly, in vitro studies showed an upregulation of YAP expression and its nuclear translocation, a downregulation of p-YAP phosphorylation and an increase in TEAD2 expression that was dose-dependent. Incubation of PC12 cells with extract alone also inhibited the increase in p-MST1(Ser410) and p-LATS1/2(Ser909/872) expression, the upregulation of Survivin and the downregulation of PML. Extract treatment also promoted a dose-dependent increase in Bcl-2/Bax ratio and a decrease in caspase 3 expression, suggesting that extract control of the neuronal cellular apoptosis may occur, at least in part, via regulation of Hippo/YAP signaling. These findings are in line with a recent study reporting that Aβ sequesters YAP from the nucleus into cytoplasmic Aβ aggregates ultimately impairing its function and leading to necrosis [62]. Treatment of cells with extract attenuated these alterations as well as the increase in caspase 3 expression levels induced by Aβ_1–42_. Inhibition of YAP expression using the inhibitor Verteporfin or CRISPR Cas9 approaches prevented the protective action of extract against Aβ_1–42_-induced damage. Further studies showed that extract treatment attenuated Aβ_1–42_-induced phosphorylation of YAP, the inhibited p-MST1(Ser410) and p-LATS1/2 (Ser909/872) expression, the reduced expression of TEAD2 and Survivin and the increased the expression of PML, further validating the involvement of YAP signaling in artemisia annua *A. annua* action.

It has been documented that YAP signaling in Hippo pathway drives TEAD regulation of the senescence and numbers of neural progenitor cells [63]. These cellular behaviors play a key role in the maintenance of the neural network activity, suggesting the Hippo-pathway as a potential novel target to treat neurodegenerative diseases such as AD. Surprisingly, in vivo results showed an increased survival and proliferation of NP cells in the cerebral cortex and hypothalamus of animals treated with *A. annua* extract. The occurrence of neurogenesis impairment has been implicated in AD [64,65], suggesting the use of therapeutic approaches able enhance adult neurogenesis as potential treatment strategies [66]. Besides its neurogenic effect, extract also attenuated the occurrence of dendritic loss by promoting the upregulation of synapse-related proteins expression. Taking these findings into account, we might speculate that the strong neuroprotective effect exerted by *A. annua* extract is due to its dual function in attenuating AD pathological alterations and promoting neurogenesis. Importantly, this action may be a result of the synergistic action of extract main active components deoxyartemisinin and arteether. 

## 4. Materials and Methods

### 4.1. Extract from Artemisia annua Preparation

Dried extract from *A. annua* were obtained from Nanjing Puyi Biotechnology company (Nanjing, China). The extraction process was performed as follows: water-soluble *A. annua* extracts were obtained by soaking *A. annua* dried powder in distilled water (1:10) wt./vol for 2 h and then they were heated and boiled for an additional 2 h (primary extraction). The extract was then filtered, soaked again in distilled water (1:8) wt./vol and left boiling for 1.5 h (secondary extraction). After filtration, the extracting solutions were evaporated at 65 °C under reduced pressure (−0.08 MPa), followed by lyophilization to obtain a relative density of 1.12.

### 4.2. Component Analysis of Extract Powder with HPLC-MS

Analytical grade standards including artemisinin, artemisinin acid, arteether and deoxyartemisinin were purchased from Meilunbio (Dalian, China). The purified water used for the HPLC-MS system was obtained from the Invitrogen company (Cat No.: 10977-023). LC/MS grade formic acid and ammonium acetate were obtained from Thermo Fisher Scientific Ltd. (Cat No.: A117-50). The acetonitrile and methanol used in the method were of HPLC grade and obtained from the Duksan Company (Cat No.:3040-4L; Cat No.:3041-4L, Seoul, Republic of Korea). Two mg of extract powder was accurately weighed and mixed with one mL of methanol in an ultrasonic bath. The obtained extract solution was then filtered by a 0.22 μm microporous membrane for HPLC-MS/MS analysis. The analysis was performed using an HPLC system coupled with an electrospray ionization (ESI) source that was operated in positive ionization mode with an Agilent 1200 HPLC system. All analytes were quantitated in the ion multiple reaction monitoring (MRM) mode. Chromatographic separation was carried out on a Sonoma C18(2) (3 u 100 A 150 mm × 2.1 mm) column. The column temperature was maintained at room temperature, and the injection volume was 5 μL. The mobile phase consisted of (A) methanol with 0.1% formic acid and (B) water with 0.1% formic acid with a flow rate of 0.4 mL/min. A gradient program was used as follows: 0–2 min 95% of A, 2–12 min 40% of A, 17 min–21 min 29% of A, 21–25 min 10% of A, 26–27 min 95% of A and 27–30 min 95% of A. All procedures were performed by Prof. Hua Yu from State Key Laboratory of Quality Research in Chinese Medicine, Institute of Chinese Medical Sciences, University of Macau.

### 4.3. Animals and Treatment

3xTg transgenic mice (APPSWE, TauP301 L and PS1M146V) were obtained from Jackson Laboratory and bred in the animal facility of the University of Macau. All animal experiments were approved by the University of Macau Animal Ethics Committee (protocol No. UMAEC-001-2020). The animals were housed in a 24–26 °C room with a 12 h/dark–light cycle and food and water were available ad libitum. Animals were randomly divided into four groups: Wild-type (WT, Ctrl), 3xTg (Ctrl), 3xTg + 6.7 mg/mL extract and 3xTg + 20 mg/mL extract groups (*n* = 10 animals per group, female, ~30 g, aged 9 months). Animals from 3xTg + 6.7 mg/mL group and 3xTg + 20 mg/mL group were treated with extract dissolved in the drinking water. Animals from the WT and 3xTg groups received equal amounts of water. After 3 months of treatment, the behavioral performance of all mice was examined by Morris water maze.

### 4.4. Behavioral Tests

The Morris water maze (MWM) test was used to analyze the effect of the extract on the memory and learning abilities of mice according to previously described methods [50,67,68]. Briefly, the animals were submitted to a place navigation test during 5 consecutive days, followed by a spatial probe trial on the sixth day. During the place navigation tests the platform was placed in the middle of one of the quadrants and 1 cm above the water surface. The path distance and latency of finding the platform were tested and used as indicators of the mice learning ability. On the day of spatial probe trial, the mice searched the platform for 60 s, which had been previously removed. The time spent in the target quadrant and the number of crossings was measured. All data acquisition and processing were performed by image analyzing software (ANY-maze; Stoelting). Before the Morris water maze test, the swimming abilities of all mice were assessed and mice unable to swim were excluded from the study.

### 4.5. BrdU Labeling and Tissue Sample Preparation

After treatment, all mice were injected with 5-Bromo-2′-deoxyuridine at a concentration of 50 mg/kg (BrdU, Sigma-Aldrich, St. Louis, MO, USA) by intraperitoneal injections administered twice a day for 4 days [69]. All mice were then euthanized using chloral hydrate (0.25 mg/mL) and the brains were dissected and fixed in 4% paraformaldehyde (4% PFA) for 48 h at 4 °C. After fixation, some of the samples were dehydrated and embedded in OCT, and kept at −20 °C until further analysis. Other samples were put into 75% alcohol, dehydrated and embedded with paraffin (Leica, EG1150, Wetzlar, Germany), and kept at 4 °C until further analysis.

### 4.6. Immunofluorescence Analysis

For immunofluorescence, the brains were cut into 20 μm slices using a low-temperature thermostat. After incubation with 0.2% Triton X-100 (Thermo Fisher Scientific, 85111, Waltham, MA, USA) for 30 min, the sections were washed with PBS and incubated with blocking buffer composed of 3% BSA (Sigma-Aldrich, A9647) in PBS for 30 min at room temperature. The samples were then incubated with primary antibodies overnight at 4 °C. On the next day, the samples were rinsed with PBS and incubated for one hour at room temperature with the corresponding secondary antibody (Alexa Fluor 488 anti-mouse or 594 anti-rabbit (1:500) (Invitrogen, A30629 and A30678, Waltham, MA, USA). The sections were blocked by anti-fluorescence quenching blocking solution with DAPI, then analyzed and photographed using a microscope (Carl Zeiss Confocal, LSM710, Oberkochen, Germany). The number of cells was counted using Image J software. The percentage of positive cells was calculated from the total nucleus population. All studies were performed three times, with 10 animals in each group. The information of all antibodies is shown in Table 1.

### 4.7. Cells Culture and Treatments

Primary neuronal cells were isolated from C57BL/6 mice brains and cultured in neuronal culture medium, as previously described [70,71]. Briefly, newborn C57BL/6 mice, obtained from the animal facility of the University of Macau, were sacrificed and the brain was surgically removed and washed with cold PBS to remove all the blood. Brain homogenates were digested with 0.25% trypsin for 10 min at 37 °C. The obtained cell suspension was filtered through a 0.45 μm pore size filter unit, centrifuged at 1000× *g* for 5 min and the supernatant removed. The obtained cells were seeded in poly-D-lysine plates with neurobasal medium containing 1% B27, 1% N2, 1% NEAA and 50 μM glutamine.

### 4.8. CRISPR/Cas9 Genome Editing

(CRISPR)/CRISPR-associated protein 9 (Cas9)-mediated gene editing was performed as previously described [72]. The sgRNA targeting YAP was obtained from Mouse_GeCKOv2_Library_A as follows: CACCGCCCAAGTCCCACTCGCGAC. The sgRNA oligo primer was synthesized by The Beijing Genomics Institute (BGI, Shenzhen, China). After that, sgRNA was cloned into the pSpCas9(BB)-2A-Puro (PX459) V2.0 sequence from Addgene company (plasmid #62988, Addgene, Watertown, MA, USA). The presence of the insert was verified by isolating the plasmid DNA from several bacterial colonies and performing sequencing from the U6 promoter (human) (CCGTAACTTGAAAGTATTTCG). Isolated plasmid from positive colonies was transfected into PC12 cells using lipofectamine™ 3000 transfection reagent according to the manufacturer’s instructions (Thermo Fisher, L3000015). The cells were sorted out using 2.5 μg/mL puromycin and cultured into single colonies in 96-well plates. YAP expression in WT and KO cells was assessed by western blot analysis [73].

### 4.9. MTT Assay

The cell viability was assessed using MTT assay as previously described [74,75]. Briefly, the cells were seeded in 96-well plates at a density of 5 × 10^3^ cells/well in complete medium. On the following day, seeded cells were incubated for 24 h with (1) different concentrations of extract; (2) different concentrations of Aβ_1–42_ and (3) Aβ_142_ with or without different concentrations of extract. After the treatment, cells were further incubated with MTT (0.5 mg/mL) for an additional 3–4 h, and the medium was replaced with 100 μL DMSO to dissolve the blue formazan crystals formed by live cells. The absorbance was measured at 570 nm using a microplate reader (SpectraMax 250, Molecular Device, Sunnyvale, CA, USA). Cell viability was calculated as a percentage of the control group. The detail information of Aβ_1–42_ was obtained from ONTORES biotechnologies company, and detailed information is provided in Table 2.

### 4.10. Measurement of Reactive Oxygen Species (ROS)

The levels of intracellular ROS were tested using the fluorescent probe DCFH-DA (Beyotime, S0033S, Nantong, China), according to the protocol provided by the manufacturer. After appropriate treatment, the cells were kept in the dark with DCFH-DA reagent (10 μM) in DMEM (without FBS) for 30 min and washed twice with phosphate-buffered saline (PBS) solution. The fluorescence was measured with an Infinite M200 PRO Multimode Microplate using 488 nm excitation wavelength and 525 nm emission wavelength.

### 4.11. Measurement of Mitochondrial Membrane Potential (Δψm)

The mitochondrial membrane potential (Δψm) was measured by mitochondrial membrane potential assay kit with JC-1 (Beyotime, C2006), according to the protocol provided by the manufacturer. After appropriate treatment, the cells were incubated with 1x JC-1 (10 μg/mL in medium without FBS) at 37 °C for 30 min and washed two times with PBS solution. The intensities of red fluorescence (excitation 560 nm, emission 595 nm) and green fluorescence (excitation 485 nm, emission 535 nm) were measured using an Infinite M200 PRO Multimode Microplate. The ratio of JC-1 red/green fluorescence intensity was used to calculate the Δψm. All the values were normalized to the control group.

### 4.12. Caspase 3 Activity Assay

The levels of caspase 3 were measured using a caspase 3 activity assay kit (C1115, Beyotime Institute of Biotechnology, Shanghai, China). After 24 h of treatment, the cells were digested with 0.25% trypsin at 37 °C for 1 min, collected and centrifuged at 500× *g* for 5 min at 4 °C. The supernatant was then removed and the cells were washed once with PBS solution. Following the manufacturer’s instructions, 100 μL of lysis buffer was added per two million cells. Cells were then lysed for 15 min on ice followed by centrifugation at 12,000× *g* for 15 min at 4 °C. Afterwards, 40 μL of the detection buffer and 10 μL of Ac-DEVD-pNA (2 mM) were added to 50 μL of each sample. Obtained solutions were mixed carefully, avoiding the production of bubbles, and incubated for 60–120 min at 37 °C. The production of pNA was measured by the absorbance values at 405 nm using an Infinite M200 PRO Multimode Microplate. Caspase-3 activity results were normalized to the control group.

### 4.13. TUNEL Assay

TUNEL staining was used to test cellular apoptosis according to the instructions provided by the manufacturer (C1090, Beyotime, Shanghai, China). Briefly, after appropriate treatment, the cells were washed two times with PBS and fixed in 4% paraformaldehyde (PFA) for 30 min. The cells were then washed one time with PBS, incubated with 0.3% Triton X-100 in PBS for 10 min at room temperature and rinsed once with PBS, followed by incubation with 0.3% H_2_O_2_ in PBS for 30 min. After this period the cells were incubated with 50 μL of TUNEL reaction mixture (5 μL of TdT enzyme and 45 μL of fluorescent labeling solution) for 60 min at 37 °C protected from light. TUNEL-positive cells (green fluorescence) were observed under a fluorescent microscope and counted. The apoptosis was calculated as a percentage of the total number of cells. For tissue samples the same methodology was used.

### 4.14. Flow Cytometry

Flow cytometric assay was performed following the instructions provided by the Sangon Biotech manufacturer (Sangon Biotech, E606336, Shanghai, China). Briefly, after appropriate treatment the cells were harvested and centrifuged at 1000 rpm for 5 min. The cells were rinsed twice with ice-cold PBS and resuspended in Annexin V-FITC/PI binding buffer (195 μL). Annexin V-FITC (5 μL) was added and the cells were kept in the dark at room temperature for 30 min. Cells were then centrifuged at 1000 rpm for 5 min and re-suspended in Annexin V-FITC/PI-binding buffer (190 μL). Propidium iodide (PI) (10 μL) was further added and allowed to incubate in the dark for 5 min. The quantification of apoptotic cells was performed using flow cytometry analysis.

### 4.15. Western Blotting

Western blot was performed to assess the expression levels of molecules or enzymes involved in Aβ production and degradation, Tau phosphorylation, oxidative stress, synapse-related proteins, apoptosis-related proteins and Hippo pathway-associated proteins. Protein samples from cultured cells and brain homogenates were successfully extracted with RIPA buffer and quantified using the BCA assay kit (Thermo Fisher, 23225). After electrophoresis, the proteins were transferred to 0.22 μm PVDF membranes for 1.5 h. The blotted PVDF membranes were blocked for 2 h with 5% BSA (TBST) and incubated with the primary antibodies (1:1000) at 4 °C overnight. After washing with TBST (3 times, 10 min each), the membranes were incubated for 2 h with the secondary antibody (1:5000) at room temperature. Enhanced chemiluminescent was used to detect the blots. All antibodies are listed in Table 1.

### 4.16. Statistical Analysis

Statistical analysis was performed using GraphPad Prism 88.0.1(244) software. All results are expressed as the mean ± SEM from three experiments. The statistical significance between multiple groups was determined using one or two-way ANOVA followed by Tukey’s post-hoc test. *p* < 0.05 was considered statistically significant.

## 5. Conclusions

**Figure 11:**
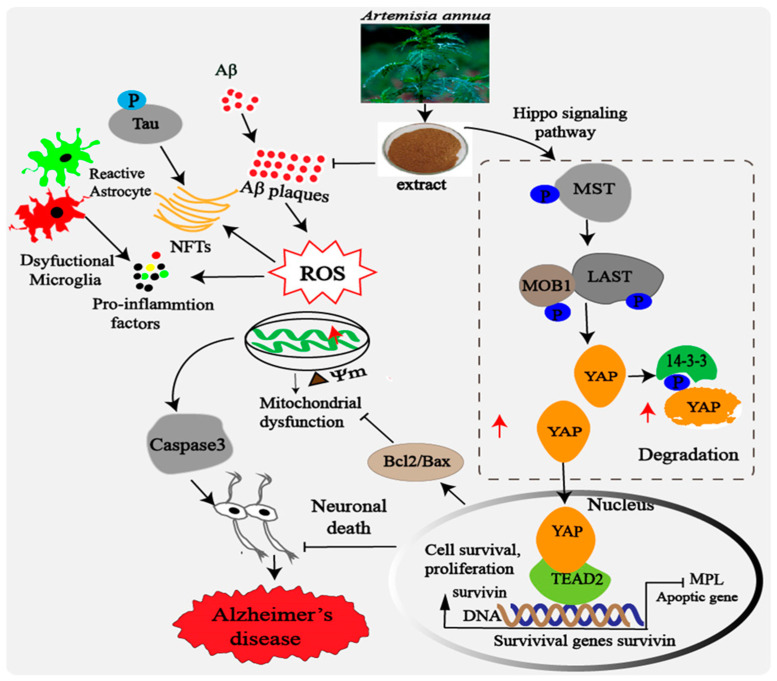
The possible mechanism of *A. annua* action on Alzheimer’s disease. *A. annua* stimulated YAP overexpression in neuronal cells and in the brain of 3xTg mice, resulting in the activation of YAP, the Survivin/Bcl2 survival pathway and inhibition of the apoptosis pathway. This process may inhibit Aβ aggregation and the downstream pathologic events triggered by Aβ such as oxidative stress, mitochondrial dysfunction, Tau phosphorylation and neuroinflammation. *A. annua* may rescue neuronal loss and prompt the functional recovery of AD through YAP/TEAD2/Survivin.

Our findings demonstrated that *A. annua* extract can improve the cognitive deficits and reverse the pathological changes of AD in vivo. In addition, *A. annua* extract has potential neuroprotective effect on Aβ_1–42_-induced neurotoxicity, characterized by reversed Aβ_1–42_-induced increase in ROS levels, caspase-3 activity, neuronal cell apoptosis in vitro. Our study further clarified the underlying therapeutic mechanisms of extract in vivo and in vitro. Although further researches are needed to elucidate the underlying beneficial ingredients of extract, we suggest the potential use of extract in AD therapy. Being able to target multiple key cascades leading to AD pathogenesis, including Aβ aggregation, tau phosphorylation, oxidative stress, mitochondrial dysfunction and neuroinflammation, *A. annua* holds great promising as a potential compound to be used in the development of novel AD therapies (Figure 11).

## 6. Limitations of This Study

In the present study, we demonstrated that *A. annua* has a very promising effect in treating AD. However, our research might have some possible limitations. First, it is well established that as a Chinese herb medicine, *A. annua* contains several biologically active ingredients, such as sesquiterpene lactones, flavonoids, polysaccharides, coumarins, volatile oils and phenolic compounds known as phenols. In this study, we only tested the therapeutic effect of total *A. annua* extract. Considering the complexity of the drug composition, we need to further identify all the beneficial components of the extract via analysis of Chinese medicine ingredients. In future research, we may consider to choose a single compound from the extract to test its effect separately. Second, we consider the use of *A. annua* to develop new products for treating or preventing AD. Therefore, the possible toxicity of *A. annua* extract, or treatment benefits, should be further evaluated in healthy animals. Lastly, considering that the pathogenesis of AD is complex and multifactorial, we should further explore how the extract affects the disappearance of oligomers or plaques of amyloid-beta in the brain of AD mice model. In this study, we only adopted Aβ_1–42_ as a marker for Aβ pathology. Future research should also consider using other types of Aβ protein to explore possible differences on the extract’s effect.

## Figures and Tables

**Table 1:** Antibody information.

Antibodies	Cat.No	Company
APP/β-Amyloid (NAB228)	2450	CST
β-Amyloid (1-42 Specific) (D3E10)	12843	CST
Phospho-Tau (Thr181)	D9F4G	CST
Anti-Tau (phospho S396)	ab109390	Abcam
β-Amyloid, (6E10)	803004	BioLegend
Tau (Tau46)	4019	CST
Iba1	GTX1004	GeneTex
GFAP (GA5)	3670	CST
SOX2	MAB5603	Millipore
BrdU (Bu20a) mouse mAb	5292	CST
IL6	32064	SAB
IL-1beta (3A6)	12242s	CST
TNF-α (D2D4) XP^®^ Rabbit mAb	11948	CST
Anti-SYP/Synaptophysin Antibody	BA3279	Boster
PSD95 (D27E11) XP^®^ Rabbit mAb	3450	CST
Bax Antibody	2772s	CST
Bcl-2 (D17C4) Rabbit mAb	3498s	CST
Cleaved Caspase-3	9661s	CST
Caspase-3 (8G10) Rabbit mAb	9665	CST
p-YAP(Ser127)	4911s	CST
YAP antibody	SC-101199	Santa Cruz
TEAD2 Antibody	33900	SAB
Survivin Antibody	24092	SAB
PML Antibody	32211	SAB
MST1 (Phospho-Thr183) Antibody	12144	SAB
LATS1/2(Phospho-Ser909/872) Antibody	12514	SAB
Phospho-MOB1 (Thr35) (D2F10) Rabbit mAb	8699	CST
MOB1 (E1N9D) Rabbit mAb	13730	CST
GAPDH	21612	SAB

**Table 2:** Detailed information of Aβ_1–42_.

Aβ_1–42_	Detail Information
Sequence (Three-Letter Code)	H–Asp–Ala–Glu–Phe–Arg–His–Asp–Ser–Gly–Tyr–Glu -Val–His–His–Gln–Lys–Leu–Val–Phe–Phe–Ala–Glu–Asp -Val–Gly–Ser–Asn–Lys–Gly–Ala–Ile–Ile–Gly–Leu–Met -Val–Gly–Gly–Val–Val–Ile–Ala–OH
One Letter Code	DAEFRHDSGYEVHHQKLVFFAEDVGSNKGAIIGLMVGGVVIA
Molecular Formula	C_203_H_311_N_55_O_60_S
Relative Molecular Mass	4514.10

## Data Availability

All data in this study are available from the corresponding author on reasonable request.

## Supplementary Materials

The following supporting information can be downloaded at: https://www.mdpi.com/article/10.3390/ijms24065259/s1.
