# Supplementary material for: Artemisia annua Extract Improves the Cognitive Deficits and Reverses the Pathological Changes of Alzheimer’s Disease via Regulating YAP Signaling
Source: Int J Mol Sci. 2023 Mar 9;24(6):5259. doi: 10.3390/ijms24065259 (PMC10049624; doi:10.3390/ijms24065259)
Supplement: Supplementary file 1 [file ijms-24-05259-s001.zip › ijms-2153723-supplementary.pdf]

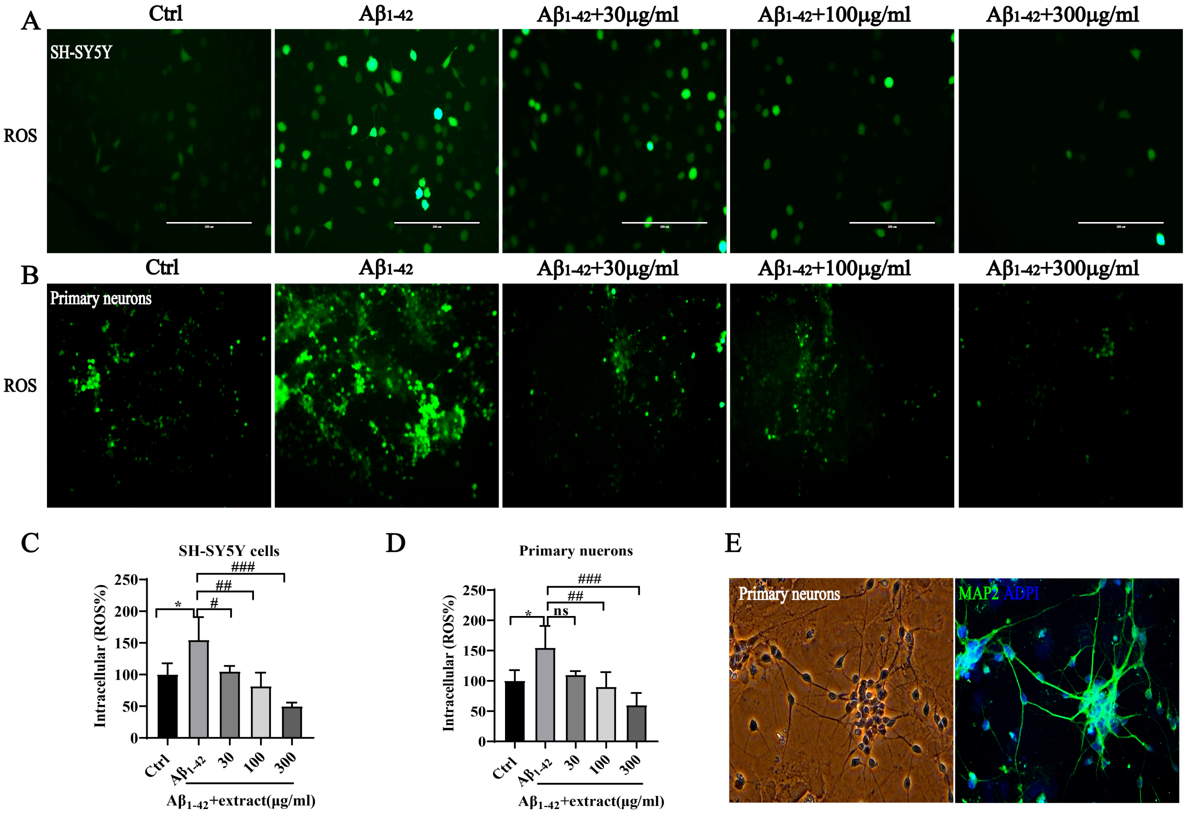

Figure S1. *A. annua* extract inhibited the increase in intracellular ROS levels promoted by Aβ1-42 in SH-SY5Y cells and primary neurons. (**A**,**B**) Representative images of ROS staining in cultured SH-SY5Y cells and primary neurons treated with 8 μM Aβ1-42 alone or with (30 μg/mL, 100 μg/mL, 300 μg/mL) extract. (**C**,**D**) Quantification of the ROS levels in SH-SY5Y cells and primary neurons. (**E**) Representative image of primary cortical neurons isolated from newborn C57 mice brain cultured for 7 days. (* $p < 0.05$; # $p < 0.05$, ## $p < 0.005$ and ### $p < 0.0005$ were considered statistically significant. * Representative comparison between Ctrl and Aβ1-42, # representative comparison between Aβ1-42 and extract; ns was not considered significantly different).

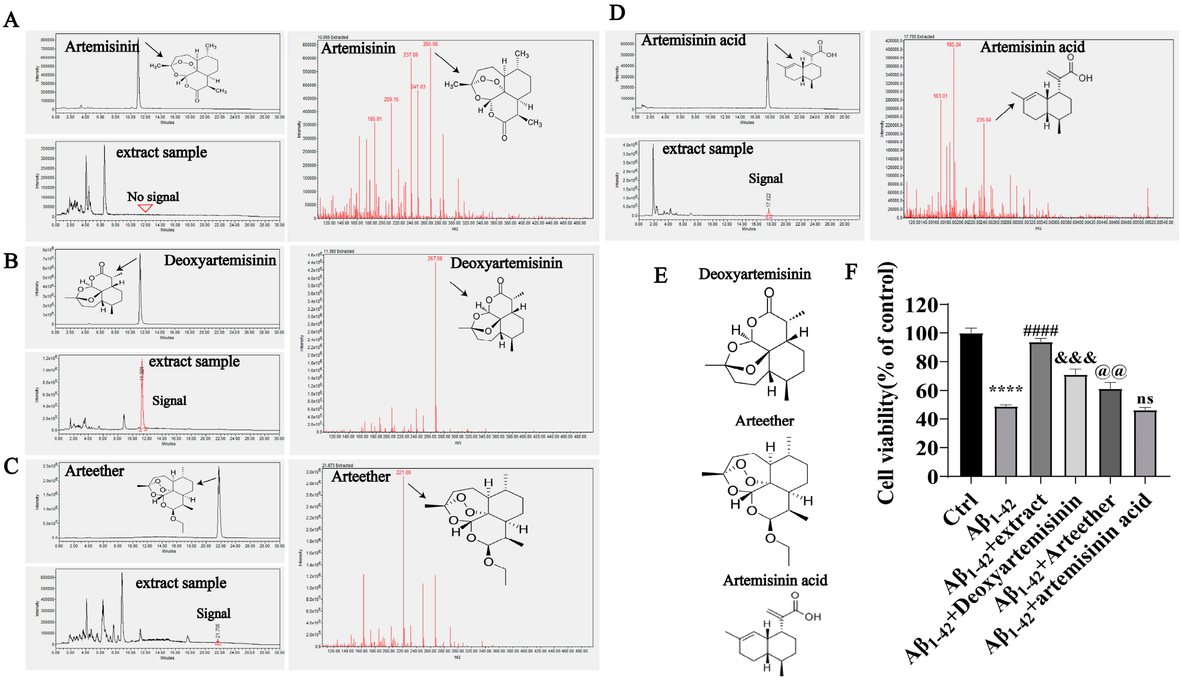

Figure S2. HPLC chromatogram analysis of *A. annua* extract. (**A–D**) Chemical standardization of extract analyzed by HPLC. Artemisinin (**A**), Deoxyartemisinin (**B**), Arteether (**C**) Artemisinin acid (**D**), and were identified. (**E**) The chemical structure of deoxyartemisinin, arteether and artemisinin acid. (**F**) Comparison of neuroprotective effects of different components by MTT assay. (**** $p < 0.0001$. *Representative comparison between Ctrl and Aβ_{1-42}; #### $p < 0.0001$#Representative comparison between Aβ_{1-42} and extract, &&& $p < 0.0005$. Representative comparison between Aβ_{1-42} and Aβ_{1-42}+deoxyartemisinin; @@ $p < 0.005$. Representative comparison between Aβ_{1-42} and Aβ_{1-42}+Arteether, were considered statistically significant.).
